# A Potential Biomarker for Acute Kidney Injury in Preterm Infants from Metabolic Profiling

**DOI:** 10.4172/2155-9929.S3-001

**Published:** 2012-02

**Authors:** Lindsey E. Romick-Rosendale, Kurt R. Schibler, Michael A. Kennedy

**Affiliations:** 1 Department of Chemistry and Biochemistry, Miami University, Oxford, OH 45056, USA; 2 Perinatal Institute, Cincinnati Children’s Hospital Medical Center, Cincinnati, OH 45229, USA

**Keywords:** Acute kidney injury, Preterm infant, Necrotizing enterocolitis, NMR, Metabonomics, Neutrophil gelatinase-associated lipocalin (Ngal), Carnitine, Blood Urea Nitrogen (BUN)

## Abstract

**Background:**

Currently used biomarkers for acute kidney injury (AKI), namely Ngal, SCr, and BUN, are inadequate for timely detection of AKI in preterm infants.

**Methods:**

Nuclear magnetic resonance (NMR) spectroscopy-based metabolic profiling was conducted on urines from 20 preterm infants to determine if novel metabolic biomarkers could be identified for early detection of AKI. Urines were collected from every patient each day for the first 14 days of life. NMR spectra were measured for all urines and metabolic profiling analysis conducted.

**Results:**

One metabolite, carnitine, increased significantly in urines of three extremely low birth weight (ELBW) infants starting on day five of life. The three affected infants either received prolonged antibiotic treatment, extended treatment with indomethacin, or both. One ELBW patient who received both treatments and reached the highest urinary carnitine level died on day 10 of life due to localized gut perforation complicated by suspected AKI.

**Conclusions:**

It was concluded that carnitine increased in the three neonates in part due to antibiotic- and/or indomethacin-induced AKI. It is hypothesized that combined antibiotic and indomethacin treatment promoted AKI resulting in reduced proximal renal tubule reabsorption of carnitine and that β-lactam antibiotics blocked renal carnitine uptake by human organic cation transporter, hOCTN2.

## Introduction

The survival rates of preterm infants, particularly those in the very low birth weight (VLBW) population born weighing less than 1500 g, has continued to increase dramatically in recent years due to improved neonatal care, advances in medical instrumentation and administration of more effective medications. However, similar advances in the treatment and diagnosis of acute kidney injury (AKI) in premature infants have yet to be seen. In a recent neonatal study, the incidence of AKI in hospitalized newborns ranged from 8 to 24% [1]. The study found that factors such as VLBW, patent ductus arteriosus accompanied by administration of antibiotics and nonsteroidal anti-inflammatory drugs, and a low Apgar score, were all associated with progression of AKI [1]. Neonates and infants, aged one day to one year, accounted for 57% of the total non-survivor group of individuals afflicted with AKI [2]. An increase in the survival rates of preterm infants has also resulted in an increase in the incidence of AKI in this patient population. AKI, previously called acute renal failure, is clinically recognized by an abrupt reduction in renal function or urine output [2]. The term AKI was chosen to reflect the full spectrum of acute kidney failure, reflecting that a sharp decline in kidney function is often a result of injury that causes functional or structural modifications in the kidneys [3].

The most common form of AKI in hospitalized patients is acute tubular necrosis (ATN) [4], which is caused by toxic or ischemic injury to the tubular epithelial cells resulting in tubular dysfunction [5]. Currently, clinicians diagnose AKI based on changes in serum creatinine (SCr) concentration [6] and blood urea nitrogen (BUN) levels [3]; however, changes in both SCr and BUN levels are late consequences of kidney damage [7] and are not direct markers injury itself [8]. Lavery et al. also pointed out that SCr concentrations fluctuate significantly over the first several weeks of a newborn’s life, with initial urine levels reflecting those of maternal kidney function due to placental transfer [9], further complicating the use of SCr as a potential marker of AKI in preterm infants. Renal tubule damage, as seen in cases of ATN, can be insufficient to cause a change in currently monitored parameters of kidney function such as SCr or BUN levels [10]. Failure of early diagnosis and treatment of AKI is partially due to lack of sensitive and specific renal markers to allow early detection of impending AKI [11]. Ideal biomarkers of AKI would be up-regulated soon after renal injury and would be independent of glomular filtration rate (GFR). In premature infants, extra-uterine renal development impacts the ability to accurately monitor changes in potential AKI biomarkers, which take place over the first few days or weeks of life [12], creating a further challenge in the discovery of biomarkers of AKI in this population.

The antecedents of AKI are often caused by various factors, with nephrotoxic and ischemic insults being common [4]. Many antibiotics, particularly aminoglycosides, are known to cause tubular injury in term and preterm neonates [13]. For example, gentamicin, which is commonly used to treat early bacterial infections in neonates, affects glomerular and tubular function and is a known risk factor for the development of AKI [14]. Indomethacin, which is used to treat patent ductus arteriosus, is also known to cause renal failure [15]. Furthermore, studies have also indicated that sepsis, which can be associated with localized gut perforation, continues to be a significant factor in causing AKI [4]. The various factors influencing the onset of AKI trigger the disorder via different mechanisms; however, a common pathway of ATN en route to AKI has been identified [16].

Neutrophil gelatinase-associated lipocalin (Ngal), a protein which binds siderophores (iron-chelating molecules secreted by microorganisms) [17], has previously been identified as a valuable biomarker of AKI in adult patients [18]; however, it has poorer prognostic value for AKI in children [11]. Ngal binds siderophores and inhibits growth of bacterial strains that rely on siderophores as a source of iron [19]. In adults, increased urinary and serum Ngal levels during AKI is a result of NGAL expression rapidly induced in the nephron in response to renal epithelial injury [20]. Lavery et al. found that Ngal alone is not an effective marker of AKI in VLBW infants due to overall variability in Ngal concentrations among preterm infants in comparison to other populations [9]; however, it was found that infants who did not have other clinical indicators for AKI also tested negative for AKI based on Ngal screening [9]. It was concluded that additional studies were needed to determine whether Ngal was a more specific biomarker of AKI than SCr in the VLBW class of newborns.

In the study reported here, we have further analyzed the urine samples from the study conducted by Lavery et al. [9] with the goal of identifying a metabolic biomarker of AKI that is more reliable than Ngal or SCr. The technique of nuclear magnetic resonance spectroscopy (NMR)-based metabonomics was used for metabolic profiling. NMRbased metabonomics is a powerful method for characterizing metabolic profiles of biological fluids [21,22] including urine, feces, serum, and spinal fluid and can offer information concerning the metabolic processes of organs such as the liver, kidneys, brain and stomach [23, 24]. High-field (850MHz) nuclear magnetic resonance spectroscopy (NMR)-based metabolic profiling was used to characterize urines of 17 VLBW and three extremely low birth weight (ELBW) preterm infants collected over the first 14 days of life. Significant elevation in the concentration of one metabolite, carnitine, was observed in three extremely low birth weight (ELBW) infants weighing less than 1000g. Day-to-day changes in carnitine levels of these three patients appeared much more stable compared to the corresponding changes in Ngal levels, which fluctuated dramatically in the same patients. Elevated carnitine concentrations in the three ELBW patients could be directly attributed to a dysfunction in the renal tubular epithelia, which is associated with AKI, as will be discussed in detail.

## Methods

### Study population

Samples were obtained from a study conducted at Cincinnati Children’s Hospital Medical Center (CCHMC) in which 20 premature infants with birth weights (BWs) between 500 and 1500 g were enrolled at three neonatal intensive care units in Cincinnati, Ohio (University Hospital, Good Samaritan Hospital, and Cincinnati Children’s Hospital). The Institutional Review Board at each of the three hospitals reviewed and approved the study and the informed consent documents. Infants were included if their BW was between 500 and 1500 g and parental consent was granted. Patients were differentiated into the following four BW categories 500–750, 751–1000, 1001–1250, and 1251–1500 g. Infants were excluded if a significant congenital anomaly was identified. One patient in the 500–750 BW category died on day 10. All preterm infants were on a standard continuous 20 mg/kg/day dosage of carnitine, except that two of the three ELBW infants received elevated dosages of 30 mg/kg/day and 35 mg/kg/day, however the third ELBW infant, who died on DOL 10, received the standard 20 mg/kg/day dosage.

### Sample collection

Urine was collected from diapers using a cotton ball, or bladder catheter if present, within the first 24 h of life and daily until day of life (DOL)-14. The infant’s diapers were checked every 4 h and collected urine was transferred to a refrigerator located at each study site. Urine samples were relocated to a −80° freezer within 48 h of collection.

### Preparation of biological fluids for NMR analysis

Urine samples were stored at −80°C after collection until prepared for NMR measurements. Samples were thawed on ice prior to preparation for NMR analysis. A one-milliliter aliquot of each sample was centrifuged for 10 minutes at 2655×g, and then 350 microliters of clear urine was pipetted into a 1.5 milliliter microcentrifuge tube. A volume of 350 microliters of buffer (300mM KH_2_PO_4_, 2 mM NaN_3_, 0.2% trimethylsilyl propionate (TSP) in 20% D_2_O, pH 7.4) was added to each urine sample. A volume of 600 microliters of each urine/buffer mixture was then pipetted into a 5mm NMR tube (Norell ST500-7).

### NMR data collection and processing

All NMR experiments were carried out on a Bruker Avance^™^ III spectrometer operating at 850.10 MHz ^1^H frequency and equipped with a room temperature 5mm triple resonance probe with inverse detection and controlled by TopSpin 2.1.4 (Bruker, Germany). All experiments were conducted at 298K. All data was collected using a spectral width of 20.0 ppm. Three ^1^HNMR experiments, optimized by Bruker (Bruker BioSpin, Billerica, MA) for use with metabonomic studies were run on all samples: a standard 1D presaturation (zgpr), the first increment of a 1D NOESY (noesygppr1d), and a CPMG (cpmgpr1d) experiment. All experiments included presaturation of the water peak. The transmitter offset frequency (O1) was set to 4002.80 Hz to obtain optimal water suppression. The 90° pulse width was determined for every sample using the automatic pulse calculation feature in TopSpin. All pulse widths were between 13 and 16 μs. Water suppression was achieved by irradiation of the water peak during a recycle delay of 4.0s with a pulse power level of 55.92 dB.

The zgpr experiment was used to screen samples and to assure that presaturation and shimming was sufficient for reliable data collection. To check the shimming, the full width at half height (FWHH) of the TSP peak was measured. The shimming was deemed acceptable when the FWHH was less than 0.9 Hz. The one-dimensional zgpr [1] HNMR spectra were acquired using 2 transients and 2 dummy scans, 65K points per spectrum giving an acquisition time of 1.92 s, 0.03 Hz of exponential line broadening, and a recycle delay of 4s. Once the spectrum was determined to be of acceptable quality the other two experiments were run. The first increment of the 1D NOESY experiment was collected using 16 transients with 4 dummy scans, 65K points per spectrum giving an acquisition time of 1.92 s, a mixing time of 10 ms, and apodized using a gaussian line broadening parameter of 0.01, and a 4 s recycle delay. The CPMG experiment was collected in order to filter broad peaks present in the spectrum. The CPMG experiment was collected using 128 transients with 4 dummy scans, 65K points per spectrum giving an acquisition time of 1.87 s, a T_2_ filter loop of 128 with an echo time of 1 ms, apodized using −.01 Hz of exponential line broadening, and a 4 s recycle delay. All NMR spectra were phased, baseline corrected, and corrected for chemical shift registration relative to TSP in TopSpin 2.1.1 (Bruker Biospin, Billerica, MA).

### Box and whisker plot analysis

Box and Whisker plots were generated in excel using a template provided by vertex42.com.

### Principal component analyses (PCA)

The data were subjected to multivariate statistical analysis using AMIX software version 3.9.5 (Bruker Biospin, Billerica, MA). NMR spectra were binned into 0.01 ppm-wide buckets over the region δ 10.0 to 0.2 ppm. The region of δ 4.75–5.1 was excluded from analysis to avoid effects of imperfect water suppression. Buckets with variances <5% were also excluded from PCA. Initially, unsupervised PCA was performed without consideration of class information. Rousseau et al. discusses the algorithm employed to calculate the principal components [25]. Visualization of the data was accomplished by inspection of the PCA scores and loadings plots.

### Mahalanobis distance and F-Value calculations

Mahalanobis distance calculations were performed in MatLab using a method to quantitatively and statistically analyze cluster separations in PCA scores plots of NMR data developed by Goodpaster and Kennedy [26].

### Identification of metabolites

Experimental NMR spectra were compared with those of candidate metabolites using the ChenomX NMR Suite (ChenomX Inc., Edmonton, Alberta, Canada). The ChenomX database was used to filter for resonance frequencies at chemical shifts corresponding to buckets identified as significant in the loadings plot. ChenomX database spectra for candidate metabolites were inspected to determine if peak patterns matched those observed in the experimental data. If the ChenomX database spectrum unambiguously matched a group of significant buckets identified in the loadings plot, direct assignment of the metabolite was made. In most cases, incorrect candidate metabolites could be eliminated by inspection and comparison with ChenomX database spectra due to mismatched peak patterns and/or intensities.

### Quantification of metabolites

Urine Ngal levels were determined for the previous study as described by Lavery et al.[9]. Carnitine levels were determined using the AMIX software. Specifically, an internal chemical shift and concentration standard, TSP, was added to each urine sample prior to NMR data collection. Once carnitine was identified using the AMIX software, the concentration of carnitine was determined by matching the intensity of the carnitine peaks in the database spectrum with the intensity of the observed carnitine peaks in the experimental spectrum. Therefore, the absolute concentration of the carnitine peaks was calibrated relative to the internal TSP standard.

## Results

### NMR metabonomics analysis

**Figure 1:**
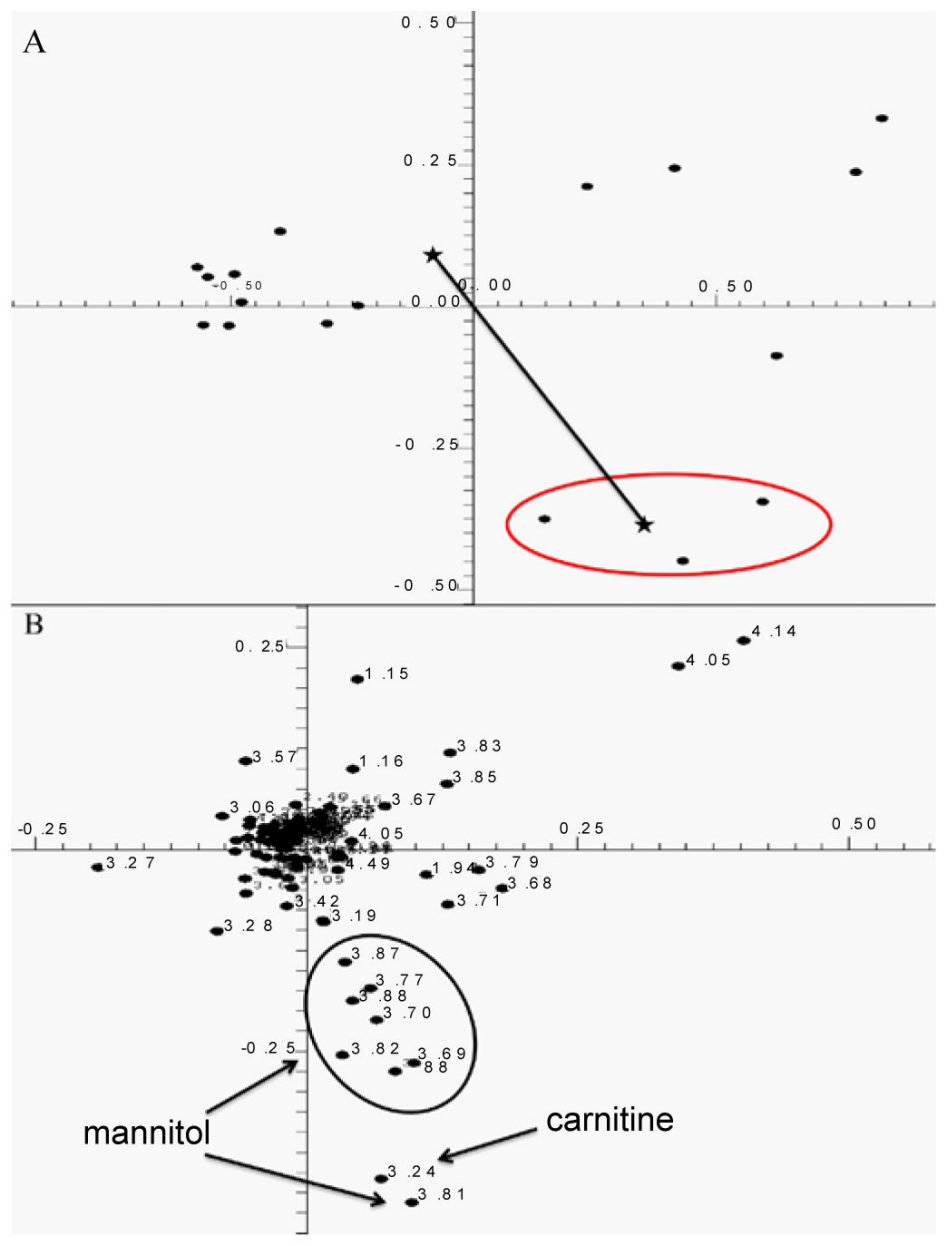
**A)** Two-dimensional scores plot of human urine samples of DOL 8 calculated using the first two principal components of the PCA. The cluster circled at the lower right represents urine samples of the three ELBW infants suffering from suspected renal injury. The stars indicate the centroids of the two clusters discussed in the text, and the line joining the centroids represents the Mahalanobis distance discussed in the text. **B)** The loadings plot corresponding to the scores plot in Figure 1A. The solid ovals encircle groups of resonance frequency buckets that belong to the same metabolite and are labeled according to the identity of the metabolite.

**Figure 2:**
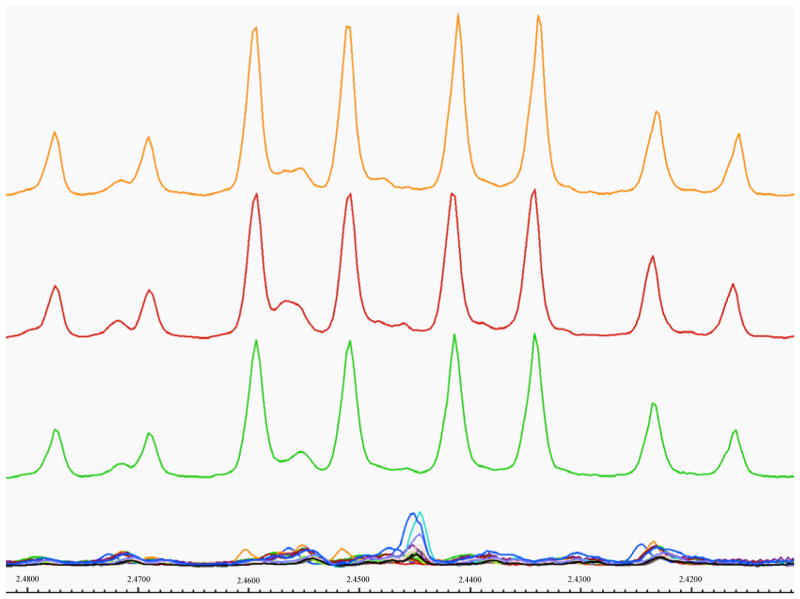
**A)** Urine NMR spectra in the region from 2.48–2.41 ppm containing a carnitine-specific multiplet for all patients on DOL 8. NMR spectra for three patients displaying elevated carnitine levels are shown at the top.

PCA of urines indicated no patient differentiation from DOL 1 – 4. Beginning on DOL 5, three patients began to group separately from the rest of the population based primarily on increased urinary carnitine (Figure 1A), which was absent in urines of all other patients. The magnitude of cluster separation was evaluated by calculating the Mahalanobis distance (D_M_) between the cluster centroids and the statistical significance of cluster separation was evaluated by calculating an F-value (Figure 1A). The D_M_ of 5.887 between carnitine-positive and carnitine-negative group centroids, and F-value of 38.394 (critical F-value 3.739), indicated statistically significant group separation. Separation of these patients based on elevated carnitine levels continued from DOL 5 until the end of the study on DOL 14. One carnitine-positive patient died on DOL 10. Visual inspection of all NMR spectra (Figure 2) confirmed that carnitine was only present in the three patients identified as outliers in Figure 1A.

**Figure 3:**
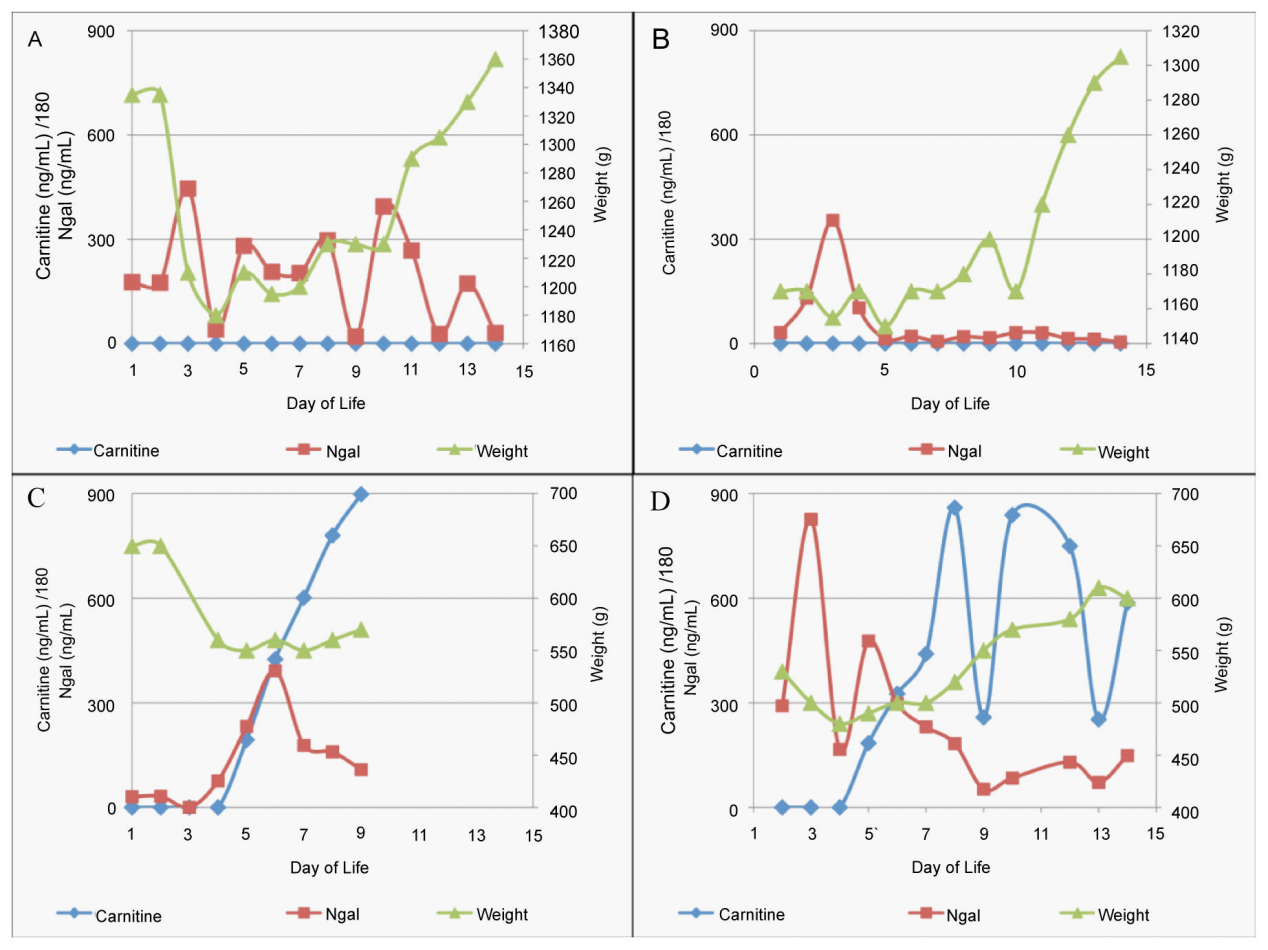
**A**–**D)** Graph of urinary carnitine and urinary Ngal levels, and correlated neonate weight. Figures 3A and 3B correspond to two carnitine-negative patients with no reported renal injury. Figures 3C and 3D correspond to two carnitine-positive patients that also had elevated urinary Ngal.

The three carnitine-positive patients were all members of the ELBW (500–750g) class of preterm infants. Figures 3A through 3D show how carnitine and Ngal levels varied with respect to body weight for two carnitine-negative patients (Figure 3A and 3B) and two carnitine-positive patients (Figure 3C and 3D). Ngal levels of carnitine-negative patients fluctuated strongly during the 14-day study (Figure 3A and 3B) while their body weights increased steadily and there were no other indications of renal injury. Figure 3C shows data for a carnitine-positive patient. The Ngal and carnitine levels began to climb on DOL 4 and DOL 5, respectively. However, whereas Ngal peaked at about 400 ng/mL on DOL 6 and then steadily decreased to ~ 100 ng/mL on DOL 9, the day before the patient’s death on DOL 10, carnitine started from 34.9 μg/mL on DOL 4 and steadily increased to 162 μg/mL on DOL 9, the day before the patient’s death on DOL 10. Based on Ngal values alone, one might conclude that this patient was moving away from indication of AKI, however carnitine steadily increased until death. This patient lost substantial body weight (~40%) just before Ngal and carnitine levels began to rise and the infant never regained significant body weight. Figure 3D shows data from another carnitine-positive patient. This patient displayed strong fluctuations in Ngal over the first week of life (ranging from <200 to >800 ng/mL) and then the Ngal levels dropped to values seen in other healthy patients. Interestingly, carnitine began to increase starting on DOL 5 reaching a maximum concentration of 151 μg/mL and fluctuated between 33.1 μg/mL and 106 μg/mL between DOL 5 and DOL 14. Over the same time period, the infant steadily gained weight resulting in a ~20% increase in body weight.

**Figure 4:**
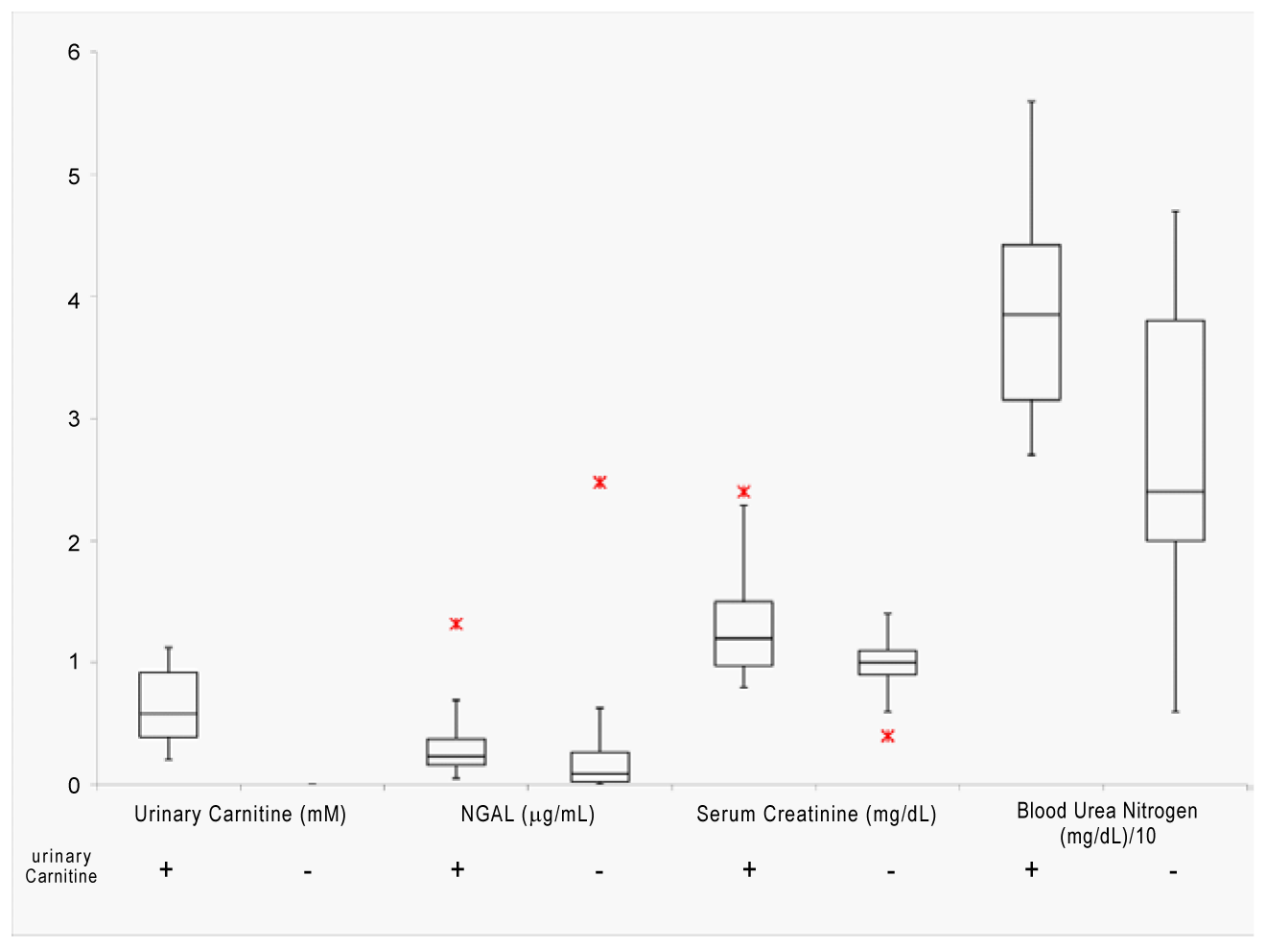
Box and Whisker plot comparing levels of urinary carnitine (mM), neutrophil gelatinase-associated lipocalin (NGAL) (μg/mL), serum creatinine (mg/dL), and blood urea nitrogen (BUN) (mg/dL) of carnitine-positive (+) and carnitine-negative (−) preterm infants.

Figure 4 illustrates how existing clinical markers of renal damage, Ngal, SCr, and BUN, varied in carnitine-positive and carnitine-negative patients from DOL 5–14. All three carnitine-positive patients presented with higher levels of all three existing renal function markers compared to carnitine-negative infants.

## Discussion

Current biomarkers for detection of AKI in premature infants, Ngal, SCr, and BUN, are inadequate [27] limited by poor sensitivity and specificity [11]. AKI is usually diagnosed indirectly by a measured rise in SCr, which is thought to indicate a reduction in GFR [28]. However, SCr concentration does not accurately mirror GFR in the non-steady-state condition of patients with AKI since SCr levels are also influenced by the degree of distribution, the rate of production, and continued renal development in preterm infants and neonates [29, 30]. Although Ngal has been identified as a useful biomarker for diagnosis of AKI in adults, the marker has not proven as useful in determining renal failure in children, and perhaps more critically, in preterm infants [9]. Ideally, biomarkers of AKI in preterm infants would present soon after renal injury and would be independent of GFR. Potential biomarkers of AKI might show up in urine during tubular inflammation or tubular dysfunction, leading to enzymes, other proteins, or small molecule metabolites in urine that are generally filtered at the glomerulus [31].

Although current prognostic indicators fall short in their ability to predict acute renal failure in its earliest stages [11, 28, 29], they are still considered the best existing measures of kidney function [3, 6, 18]. Therefore, we evaluated carnitine as a potential marker of early AKI by comparing its levels to currently used clinical biomarkers in carnitine-positive and carnitine-negative preterm infant patients. Carnitine-positive patients exhibited higher levels of Ngal, SCr, and BUN compared to the 17 carnitine-negative patients suggesting that these patients were experiencing greater renal dysfunction. Collectively, our data suggest that multiplexing a carnitine-positive measurement with other markers for AKI may lead to a more sensitive, specific, and accurate predictive capability of AKI in preterm infants.

Eighteen of twenty infants in the study, including the ELBW infant that died on DOL 10, received daily continuous dosages of carnitine of 20 mg/kg/day, while the two other ELBW infants received 30 mg/kg/day and 35 mg/kg/day; importantly, the ELBW who died on DOL 10, and who presented the highest carnitine levels, received the normal carnitine dose of 20 mg/kg/day. Carnitine levels fluctuated less than urinary Ngal in these three newborns. Under normal physiological conditions, ~95% of filtered carnitine is reabsorbed [32] and the kidneys play a fundamental role in maintaining carnitine homeostasis [33]. Metabolic equilibrium of carnitine is actively sustained by rate of biosynthesis [34], efficient reabsorption [35], and initial absorption of carnitine from dietary intake [36]. We hypothesize that urinary carnitine was increased in ELBW preterm infants due to failed or reduced proximal tubular reabsorption, a common symptom of ATN, which is known to be the final step leading to onset of AKI. Several contributing factors could explain increased urinary carnitine in carnitine-positive patients.

First, increased urinary carnitine could result from leakage from the kidneys into urine due to kidney damage associated with antibiotic treatment; specifically administration of gentamicin could have caused tubular damage and initiated AKI. Several antibiotics, predominantly aminoglycosides like gentamicin, cause renal tubular injury often characterized by tubular functional defects [37, 38]. Indeed, gentamicin is a common cause of AKI [39] and administration of gentamicin could contribute to elevated urinary carnitine due to gentamicin-induced renal injury making the renal tubules incapable of reabsorbing carnitine after filtration by the glomeruli. Without normal reabsorption into the kidneys, carnitine would be expelled as urinary waste. Gentamicin-associated carnitine loss would not only compromise the renal tubules, but might also alter normal gut function and morphology given that fatty acid oxidation in epithelial cells of the intestine and colon is facilitated by carnitine [40].

Second, increased urinary carnitine could result from a primary carnitine uptake deficiency caused either by a genetic defect in, or antibiotic-associated blockage of, human organic cation transporter (hOCTN2). hOCTN2 is a sodium-ion-dependent carnitine transporter [32] known to actively transport carnitine across membranes in renal tubules [41]. A defect in, or disruption of, hOCTN2 would result in increased urinary carnitine wasting due to defective reabsorption. Many β-lactam antibiotics, such as ampicillin, block normal transport/uptake of carnitine by competitively binding to hOCTN2 in the kidneys [42]. Because the preterm infants were administered a cocktail of antibiotics including both gentamicin and ampicillin, ampicillin binding to hOCTN2 in kidney epithelial cells could block normal carnitine uptake and transport across cell membranes, leading to increased urinary carnitine. Administration of β-lactam antibiotics could increase risk of disruption of normal carnitine uptake in kidneys and cause a general increase in urinary carnitine of affected patients.

Finally, increased urinary carnitine could have been associated with indomethacin treatment. Indomethacin, a cyclooxygenase (COX) inhibitor, is commonly administered to treat patent *ductus arteriosus* [43]. Inhibition of COX by indomethacin prevents normal decline in pulmonary vascular resistance linked to rhythmic lung distention at birth [43]. However, indomethacin also causes reduced renal blood flow [44] and almost always leads to renal failure [15]. COX inhibitors not only impair renal function by affecting renal perfusion, but also alter overall kidney development related to changes in local prostaglandin synthesis. Indomethacin has also been associated with isolated bowel perforation caused by vasoactive effects that lead to diminished mesenteric perfusion [45].

The three carnitine-positive patients were all treated either with an antibiotic cocktail containing ampicillin and gentamicin, indomethacin, or both. The carnitine-positive patient who died on DOL 10 received antibiotics for more than seven days and also received indomethacin. This patient appeared to suffer from AKI, based on elevated Ngal and carnitine levels, as well as isolated bowel perforation, which was ultimately the cause of death. We hypothesize that extended combined treatment with antibiotics and indomethacin led not only to bowel ischemia but also to AKI and renal dysfunction leading to the patient’s death. The two remaining carnitine-positive ELBW patients received antibiotics or indomethacin, but not both, which could explain fluctuations in urinary carnitine and Ngal levels as well as survival through DOL [14]. Given that all twenty infants received continuous daily therapeutic carnitine, it appears that urinary carnitine in preterm infants might be used analogously to a glucose tolerance test for diabetes, i.e. healthy preterm infants appear capable of completely absorbing therapeutic doses of carnitine, whereas infants with suspected AKI cannot. Additional studies of larger preterm infant cohorts are needed to further validate carnitine in AKI diagnosis as well as effects of antibiotics and indomethacin administration on survival rates of premature neonates.
